# Ski Skating Race Technique—Effect of Long Distance Cross-Country Ski Racing on Choice of Skating Technique in Moderate Uphill Terrain

**DOI:** 10.3389/fspor.2020.00089

**Published:** 2020-07-14

**Authors:** Luca Paolo Ardigò, Thomas Leonhard Stöggl, Tor Oskar Thomassen, Andreas Kjæreng Winther, Edvard Hamnvik Sagelv, Sigurd Pedersen, Tord Markussen Hammer, Kim Arne Heitmann, Odd-Egil Olsen, Boye Welde

**Affiliations:** ^1^Department of Neurosciences, Biomedicine and Movement Sciences, School of Exercise and Sport Science, University of Verona, Verona, Italy; ^2^Department of Sport and Exercise Science, University of Salzburg, Salzburg, Austria; ^3^Athlete Performance Center, Red Bull Sports, Salzburg, Austria; ^4^School of Sport Sciences, UiT the Arctic University of Norway, Tromsø, Norway

**Keywords:** endurance, cross country skiing, skiing technique, gradient, elite skiers

## Abstract

The aim of this study was to investigate the effect of prolonged ski racing using skating style on technique choice in a transition section among female and male high-level skiers. Fifty three national-to-elite level skiers (20 females: 26.7 ± 4.8 years, 167.0 ± 6.5 m, 61.0 ± 5.1 kg, and 75.5 ± 68.8 FIS points; 33 males: 25.2 ± 3.5 years, 179.0 ± 5.2 cm, 73.1 ± 5.7 kg, and 73.7 ± 63.2 FIS points) were video recorded along a flat-to-uphill transition section of a course during the 30-km (females) and 50-km (males) races at the 2018 Norwegian National Championships. Across laps, section speeds decreased (*P* < 0.001) in all skiers, with the best-ranked skiers faster than the lowest-ranked (*P* < 0.001), and males faster than females in the first and middle laps. Section speed within each lap was associated with race performance (*r* = 0.76–0.86, *P* < 0.001 in females and *r* = 0.87–0.89, *P* < 0.001 in males). The prevalence of Gear 2 (G2) increased, while Gear 3 (G3) use decreased (both *P* < 0.001) across the subsequent laps, with females preferring G2 more than males in lap one (*P* = 0.027). In long-distance skate-style skiing, transition performance is representative of race performance and skiers decrease the use of the often-faster G3 technique while increasing the use of the slower G2 technique due to prolonged exercise. Especially female skiers should consider adding some flat-to-uphill G3 practice into established training, specifically early in the session before fatigue may occur.

## Introduction

**Figure 1:**
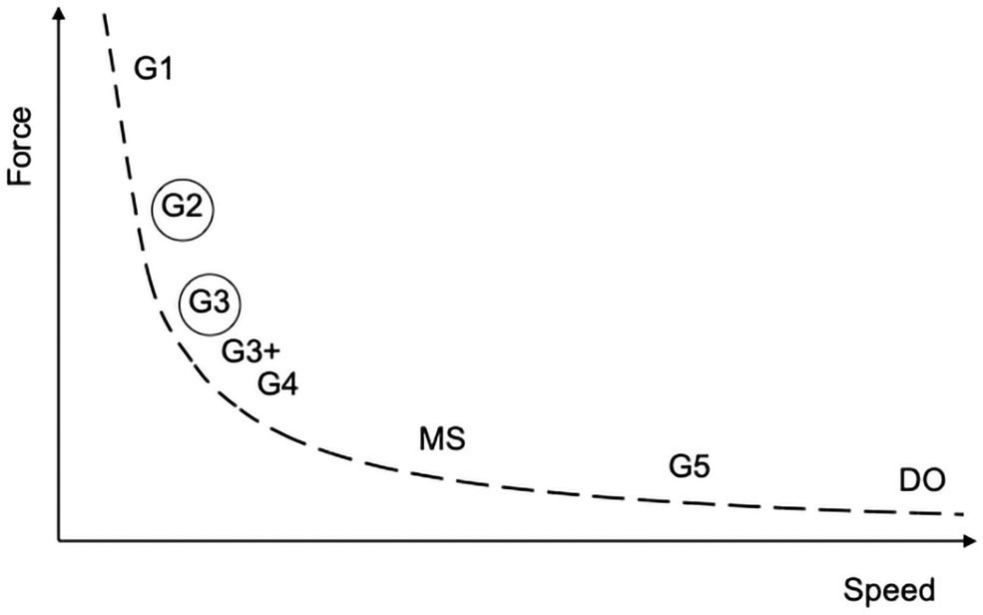
Schematic illustration of the relationship between force and speed for the eight sub-techniques of ski skating. G1 (Gear 1), G2 (Gear 2), etc. refer to the nomenclature for Swedish ski skating techniques. G3+, double-push skating; MS, marathon skating and DO, downhill. The sub-techniques circled were the *focus* of the present investigation.

Cross-country skiing (XCS) can be practiced with so-called classical style or freestyle, i.e., with skating allowed. Both the classical and skating styles consist of different techniques, developed over years and refined to specific demands of terrain within a course or race requirements in terms of force, speed, and skier capacity (Stöggl et al., 2010, 2013). It has been shown and agreed that skiers choose one technique over another to selectively develop the necessary force/speed when overtaking during a race (Losnegard, 2019). Choosing one skating technique instead of another implies repeated transitions between techniques. This is clearly an optimization phenomenon familiar in physiology and biomechanics regarding the gait of humans (Minetti et al., 1994) and other animals (Minetti et al., 1999). In ski skating, for instance, using the Swedish terminology, the so-called Gear 2 (G2) is mostly chosen to climb, whereas Gear 3 (G3) is generally preferred along moderately uphill sections and/or to increase speed (Andersson et al., 2010). At least theoretically, the skier chooses only one technique given a particular gradient/speed and while considering the metabolic power and technical skills. Other factors may influence this choice, e.g., snow or weather conditions, wind, fatigue, or subjective confidence with more “force-producing” techniques (e.g., G3 instead of the more “speed-producing” Gear 4 [G4]) during final sprints. Limited to ski skating, the relationship between force, speed, and (up to) eight different techniques is schematically shown in Figure 1. Recent articles have confirmed the above-mentioned relationship between choices of force/speed/technique in classical skiing (Dahl et al., 2017; Welde et al., 2017; Stöggl et al., 2018b). Double poling, double poling with a kick, and diagonal stride are the techniques of choice for flat, moderate, and steep uphill terrain, respectively. Despite the debatable correspondence between some classical and skating techniques aiming at similar force or speed achievement (i.e., diagonal stride and G2, and double poling and G3) (Sandbakk et al., 2015; Torvik et al., 2019), it is reasonable to believe that the relationship between choices of force/speed/technique also applies to ski skating.

In recent years there have also been some studies on race analysis (Andersson et al., 2010; Stöggl et al., 2010, 2018b; Welde et al., 2017). For instance, during simulated racing and using a combined global navigation satellite system and video analysis approach Andersson et al. (2010) found that, limited to a sprint time trial, performance was related to uphill speed, which in turn was related to G3 use. Previously, during real racing using a video recording-based approach, Bilodeau et al. (1996) found that most skiers in a 30-km race used G2 on moderate uphill terrain and G4 on flat terrain. Namely, Bilodeau et al. (1996) recorded skiers in a race consisting of two 15-km laps during the 1994 Canadian National Cross-Country Ski Championships. Skiers were investigated along two 30 m sections, 7° steep and flat, *per* lap and therefore only twice *per* incline over the whole race. However 25 years ago, skiers and equipment were generally quite different from today (Holmberg, 2015; Pellegrini et al., 2018).

Researchers have often used roller skiing as a proxy for XCS on snow, with video analysis as the most used methodology for kinematic studies of the races. However, there have been very few on-snow studies of simulated (Andersson et al., 2010; Ohtonen et al., 2018) or real races (Bilodeau et al., 1996; Marsland et al., 2017, 2018). In fact, an extensive project on the effects of real long-distance XCS competitions on biomechanical variables, including determinants of skiing technique choice, started in 2016 (Welde et al., 2017; Stöggl et al., 2018b; Jonsson et al., 2019). Kvamme et al. (2005) found that G3 (faster technique) becomes more metabolic energy-demanding than G2 (slower technique) at inclines steeper than 4–5° using the roller skiing model. However, researchers investigated a relatively low-level sample consisting of Nordic combined and biathlon athletes. Therefore, there remains a clear knowledge gap about optimal technique choice over long-distance ski skating races. Technique choice is not only relevant *per se*, but increases in relevance over the flat-to-uphill (moderate uphill) sections of courses that can be defined as “transition sections.” Such sections have already been shown to be relevant for overall long distance/multi-lap race performance, at least in the classical style (Welde et al., 2017; Stöggl et al., 2018b). Optimal technique choice may increase speed and decrease metabolic demand and fatigue. However, this has not yet been proven in skating races. This aspect gives particular relevance to optimal technique choice by racers, especially over the final kilometers of tight races.

Modern classification algorithms based on a wearable sensor have enabled novel investigations into techniques and their choice in classical skiing (Marsland et al., 2012, 2015, 2018; Rindal et al., 2017), during racing (Marsland et al., 2017), and ski skating (Myklebust et al., 2014). Wearable sensor-based analysis has thus been shown to be an alternative although less accurate approach to an analysis based on video recordings.

The main aim of the present study was to investigate the effect of prolonged ski racing using skating style on technique choice in a transition section among female and male high-level skiers over the 30-km (females) and 50-km (males) races at the Norwegian National Championships held in Alta in 2018. Sex and level effects were investigated, as well. A special case is the sex comparison, which, however, has already been extensively studied in the literature (Stöggl et al., 2018b). In our opinion, comparing the sexes in terms of performance during different physical efforts has only the limited significance of investigating the naturally different and sex-specific fatigue effects due to such efforts. It was hypothesized that (1) flat-to-uphill transition section speed would correlate with overall race speed, (2) along the transition section, skiers would only choose between G2 and G3 techniques, (3) over prolonged skiing, skiers would prefer G2 to G3, (4) there would be differences between sexes and/or performance levels (i.e., males and/or faster skiers would prefer G3 to G2 more than females and/or slowest skiers).

## Materials and Methods

### Participants

The day before the races, 37 female (of 63 registered in total) and 63 male (165 registered) skiers indicated their availability. After the races, these numbers were reduced to 20 and 33, respectively (Table 1) due to non-starters (9 women and 7 men), non-finishers, (4 men), and/or problems with the video recordings (e.g., too many skiers in the field of view, hiding one another, 8 women and 19 men). To assess differences on the basis of level of performance, both the women and men were divided into elite and national groups on the basis of their racing times *a posteriori* (Table 1). The current study was part of a larger project in which the biomechanical parameters associated with long-distance XCS during an actual skating competition are also analyzed in detail. This study was pre-approved by the Norwegian Center for Research Data and designed in accordance with the Declaration of Helsinki. All participants gave written informed consent and allowed for data usage.

**Table 1:** Participant data with statistical differences.

	**All females**	**Females elite**	**Females national**	***P***	**Cohen's *d***		**All males**	**Males elite**	**Males national**	***P***	**Cohen's *d***	
	M ± *SD*	M ± *SD*	M ± *SD*				M ± *SD*	M ± *SD*	M ± *SD*			
*n*	20	10	10				33	16	17			
Age (years)	26.7 ± 4.8	27.5 ± 4.6	25.9 ± 5.1	0.470^a^	0.33	Low	25.2 ± 3.5	26.2 ± 2.6	24.2 ± 4.1	0.104	0.57	Medium
Height (m)	167.0 ± 6.5	169.5 ± 5.5	164.5 ± 6.7	0.124^a^	0.77	Medium	179.0 ± 5.2	178.0 ± 5.1	180.0 ± 5.3	0.402	0.38	Low
Mass (kg)	61.0 ± 5.1	62.1 ± 4.6	59.9 ± 5.5	0.392^a^	0.43	Low	73.1 ± 5.7	71.8 ± 5.2	74.1 ± 6.1	0.382	0.41	Low
FIS points	75.5 ± 68.8	31.0 ± 29.8	119.9 ± 68.7	0.000^b^	1.68	Large	73.7 ± 63.2	32.30 ± 20.73	112.74 ± 65.21	0.000	1.66	Large
Race time (s)	5359 ± 434	5053 ± 127	5665 ± 416	0.000^b^	1.99	Large	8379 ± 559	7905 ± 227	8825 ± 374	0.000	2.97	Large

a *P from two-tailed, two-sample heteroscedastic Student's t-Test between elite and national level females and males, respectively*.

b *P from one-tailed, two-sample heteroscedastic Student's t-Test between elite and national level females and males, respectively*.

### Methodology

**Figure 2:**
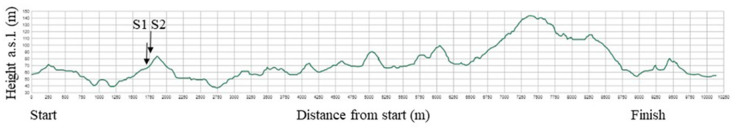
Height above sea level [a.s.l. (m)] of the 10 km ski skating cross-country race course vs. distance from start (m). Profile (m) of the 10 km ski skating cross-country race course vs. height above sea level [a.s.l. (m)]. Females covered three laps and males five laps. S1 (5.0° incline) and S2 (8.1°) indicate sections where skiers were filmed. See text for further details.

The races (individual start at 30-s intervals) consisted of three and five 10 km laps, starting at 11:30 AM and 1:30 PM, respectively. For each lap the height difference was 106 m, maximal climb 73 m, total climb 340 m, and length 10 145 m (Figure 2). On each lap, the skiers were video recorded on a 21.2 m section approximately 1.7 km after the start. This section consisted of two sub-sections, slightly overlapping: the first 10.7 m long and 6.2 m wide with an incline of 5.0° (median of three points, range 4.1–6.6°) and the second 10.5 m long and 7.5 m wide with an incline of 8.1° (five points of measurement, range 7.3–8.8°).

Two Sony Handycam FDR-AX53 video cameras (Sony Corp., Tokyo, Japan) set at 100 Hz and a shutter speed of 1/500 s recorded the skiers at 4K resolution (3840 × 2160 progressive scan). Both cameras were fixed and leveled with an electronic inclinometer on top of 1 m high tripods (SLIK Pro 700DX, Saitama, Japan) placed on custom-made wooden platforms. The cameras were positioned perpendicular to and 11 m (first sub-section) and 10 m (second sub-section) from the track, one pointing to the mid-point of each sub-section, with a focus and zoom that covered the entire sub-section. For calibration and to define the field of view, four red poles were placed at each corner of a rectangle, with a fifth pole in the middle of the side of this rectangle farthest away from the camera. All distances were carefully determined with a measuring tape. To further aid calibration and video analysis, orange fluorescent spray was used to draw two lines on the snow between the front and rear poles, perpendicular to the track. Scaling factors (real-life to video size) were applied using Kinovea software (0.8.268,9,17).

The race course was prepared with a grooming machine the previous evening and track conditions were good. Experienced technicians prepared the skis with base and high-fluorinated paraffin wax in combination with Fluor powder. Prior to the race, each skier performed her/his own personal warm-up for a 30- or 50-km ski skating race. The weather was partly cloudy with a light breeze and air temperature constantly between −2.0 and 0.0° C (relative humidity 51-54%). The snow temperature was −3.8° C at 11:30 AM, increasing gradually and slightly to −2.8° C at 3:00 PM (middle of the 50-km race), and then falling back to −3.0° C at 5:00 PM (end of the 50-km race). Gliding conditions on each sub-section were checked hourly by a ski tester using a pair of skis prepared for the conditions at 11:30 AM. Photocell gates (Brower Timing System, Salt Lake City, USA; accuracy 0.01 s) were present along a 25 m downhill section of the race track in the vicinity of the section analyzed. The gliding time remained unchanged during the entire race, with mean speeds (two runs *per* test) of 2.84 (11:30 AM), 2.83 (3:00 PM), and 2.84 m/s (5:00 PM). Race times were provided by the official timing system (EQ Timing AS, Oslo, Norway).

The section time for each lap was determined by free-application Kinovea 0.8.26 software (https://www.kinovea.org) based on when the skier passed the lines at the beginning and end of the section analyzed. Subsequently, all video recordings were examined visually to count whole skiing cycles with the different techniques. In particular, mean section speed, cycle length and rate, and technique choice were measured, calculated or assessed.

### Statistical Analysis

Results were provided as means ± SDs. A Shapiro-Wilks test revealed that the data did not significantly deviate from a normal distribution. Regarding participants' data, effect size was calculated as Cohen's *d* (0 < *d* < 0.2 *trivial*, 0.2 < *d* < 0.5 *low*, 0.5 < *d* < 0.8 *medium*, and *d* > 0.8 *large* effect). A three-way mixed repeated measures ANOVA-model with repetitions on laps (three laps; first, middle, final) as within-subject factor, and the two sexes as well as two levels of performance (elite, national) as between-subject factors, were conducted for each variable. The analyses included all the main effects and their two-way interactions. To allow for the difference in race lengths for females and males, the first, middle, and final laps (i.e., first, second, and third laps for females, and first, third, and fifth laps for males) were chosen as representative of rested, an average level of fatigue, and a high level of fatigue, respectively. When ANOVA indicated statistical significance, it was followed up with univariate ANOVA testing to identify precisely the pair-wise differences (i.e., on which of the three laps). In cases where only two means were compared (e.g., sex and performance group), independent-samples *t*-tests were performed. To determine the relationships between total and section speed, these were calculated separately for women and men using Pearson's product moment correlation. For all analyses, the level of statistical significance was set at α = 0.05. All statistical analyses were carried out using the SPSS 24.0 (SPSS Inc., Chicago, IL, USA) software.

## Results

Mean section speeds significantly decreased across laps in all skiers [Table 2; *F*_(2, 48)_ = 63, *P* < 0.001]. Sex effect was not significant (*P* = 0.118), unlike the elite-national grouping [*F*_(1, 49)_ = 59, *P* < 0.001], with elite skiers faster than national over each lap (all *P* < 0.001). Lap x sex interaction was significant [*F*_(1, 48)_ = 7.4, *P* = 0.002], with males being faster than females in the first and middle laps (both *P* = 0.006) but no difference in the final lap (*P* = 0.238). By contrast, lap x group interaction was not significant (*P* = 0.279). When considered separately for the sexes, elite skiers were faster than national (females: 1st lap +11% and 2nd and 3rd laps +12%; males: 1st lap +8%, 2nd lap +7%, 3rd lap +9%, 4th lap +15%, and 5th lap +19%).

**Table 2:** Cycle characteristics of best- (elite, *n* = 26) and worst-ranked (national, *n* = 27) male (M, *n* = 33) and female (F, *n* = 20) skiers across laps during a XC skiing race and their interactions with sex and level of performance (*n* = 53).

**Variable**		**First lap**		**Middle lap (2/3)**		**Final lap (3/5)**		**ANOVA**				
		**F**	**M**	**F**	**M**	**F**	**M**	**Lap**	**Sex**	**Group**	**Lap x Sex**	**Lap x Group**
Cycle length (m)	Total	3.83 ± 0.48^SL^		3.70 ± 0.53^SL^		3.49 ± 0.50^*^^S^^L^		*F*_(2, 48)_ = 12.5 *P* < 0.001	*F*_(1, 49)_ = 56 *P* < 0.001	*F*_(1, 49)_ = 44 *P* < 0.001	*P* = 0.090	*P* = 0.806
	Elite	3.67 ± 0.30	4.26 ± 0.33	3.59 ± 0.50	4.11 ± 0.29	3.38 ± 0.24	4.02 ± 0.34					
	National	3.23 ± 0.26	3.86 ± 0.37	3.10 ± 0.33	3.73 ± 0.47	3.18 ± 0.48	3.23 ± 0.36					
Cycle speed (m/s)	Total	4.14 ± 0.45^*^^S^^L^		3.88 ± 0.45^*^^S^^L^		3.42 ± 0.56^*^^L^		*F*_(2, 48)_ = 63 *P* < 0.001	*P* = 0.118	*F*_(1, 49)_ = 59 *P* < 0.001	*F*_(1, 48)_ = 7.4 *P* = 0.002	*P* = 0.279
	Elite	4.26 ± 0.29	4.51 ± 0.33	4.09 ± 0.23	4.25 ± 0.32	3.81 ± 0.22	3.81 ± 0.41					
	National	3.68 ± 0.40	3.99 ± 0.35	3.37 ± 0.42	3.71 ± 0.27	3.22 ± 0.42	2.94 ± 0.47					
Cycle rate (Hz)	Total	1.09 ± 0.13^S^		1.06 ± 0.12^S^		0.99 ± 0.13^*^^S^^L^		*F*_(2, 48)_ = 17.4 *P* < 0.001	*F*_(1, 49)_ = 27 *P* < 0.001	*F*_(1, 49)_ = 59 *P* < 0.001	*P* = 0.372	*P* = 0.295
	Elite	1.16 ± 0.11	1.06 ± 0.09	1.15 ± 0.13	1.04 ± 0.07	1.13 ± 0.11	0.95 ± 0.07					
	National	1.15 ± 0.15	1.04 ± 0.12	1.09 ± 0.15	1.00 ± 0.11	1.03 ± 0.14	0.91 ± 0.10					
G2 (%)	Total	36 ± 27^*^^S^		51 ± 23^*^		71 ± 22^*^		*F*_(2, 48)_ = 49 *P* < 0.001	*P* = 0.190	*P* = 0.149	*F*_(1, 48)_ = 7.4 *P* = 0.002	*P* = 0.700
	Elite	46 ± 24	24 ± 28	57 ± 21	37 ± 28	65 ± 16	69 ± 25					
	National	47 ± 30	36 ± 22	61 ± 18	56 ± 17	68 ± 23	79 ± 23					

*Total, elite and national males and females combined. All values presented are means ± SD*.

* *significantly different from all other laps*.

S *significant difference between females and males on the same lap*.

L *significant difference between elite and national skiers on the same lap*.

*NS, not significant*.

The decrease in all skiers' mean section speed was due to both a reduction in cycle length [CL; *F*_(2, 48)_ = 12.5, *P* < 0.001] and rate [CR; *F*_(2, 48)_ = 17.4, *P* < 0.001] (Table 2). Sex effect was significant regarding both CL [*F*_(1, 49)_ = 56, *P* < 0.001] and CR [*F*_(1, 49)_ = 27, *P* < 0.001], with males demonstrating longer but less frequent cycles than females over each lap. Group effect was also significant regarding both CL [*F*_(1, 49)_ = 44, *P* < 0.001] and CR [*F*_(1, 49)_ = 59, *P* < 0.001], with elite skiers using longer cycles over each lap and more frequent cycles only in the final lap. By contrast, lap x sex interaction was not significant for CL (*P* = 0.090), or for CR (*P* = 0.372). This also applied to lap x group interaction (i.e., CL: *P* = 0.806 and CR: *P* = 0.295).

**Figure 3:**
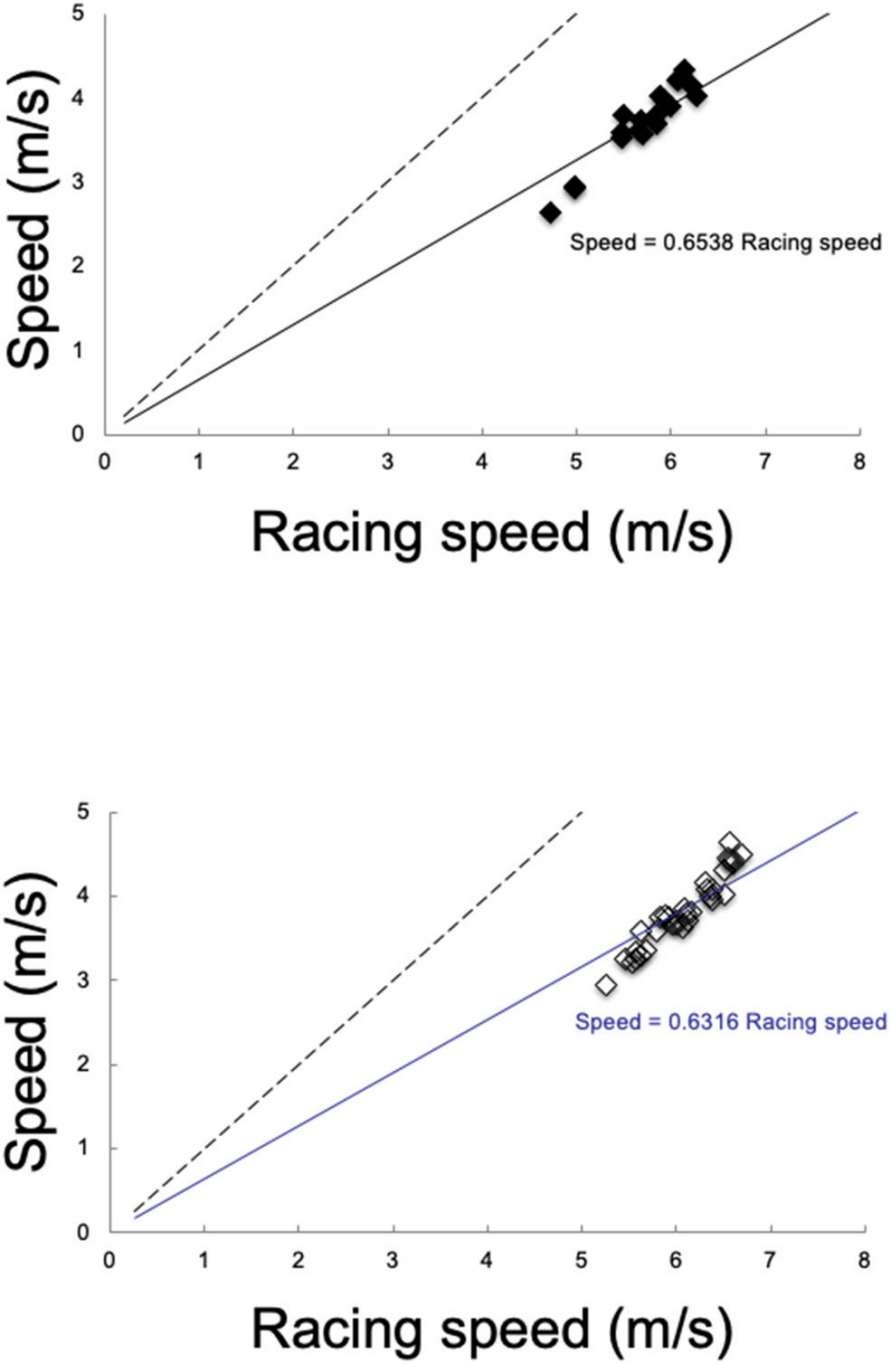
Section speed (all laps pooled) in relationship to racing speed for female (top, filled black diamonds) and male skiers (bottom, empty black diamonds). The black dotted line = line of identity. Linear regressions lines and functions through skiers values are there for indicative purpose, only.

The mean speed in the transition section for each lap was associated with race speed in both females (lap1: *r* = 0.76, lap2: *r* = 0.86, lap3: *r* = 0.85; all *P* < 0.001) and males (lap1: *r* = 0.77, lap2: *r* = 0.78, lap3: *r* = 0.81, lap4: *r* = 0.89, lap5: *r* = 0.89; all *P* < 0.001) (Figure 3).

**Figure 4:**
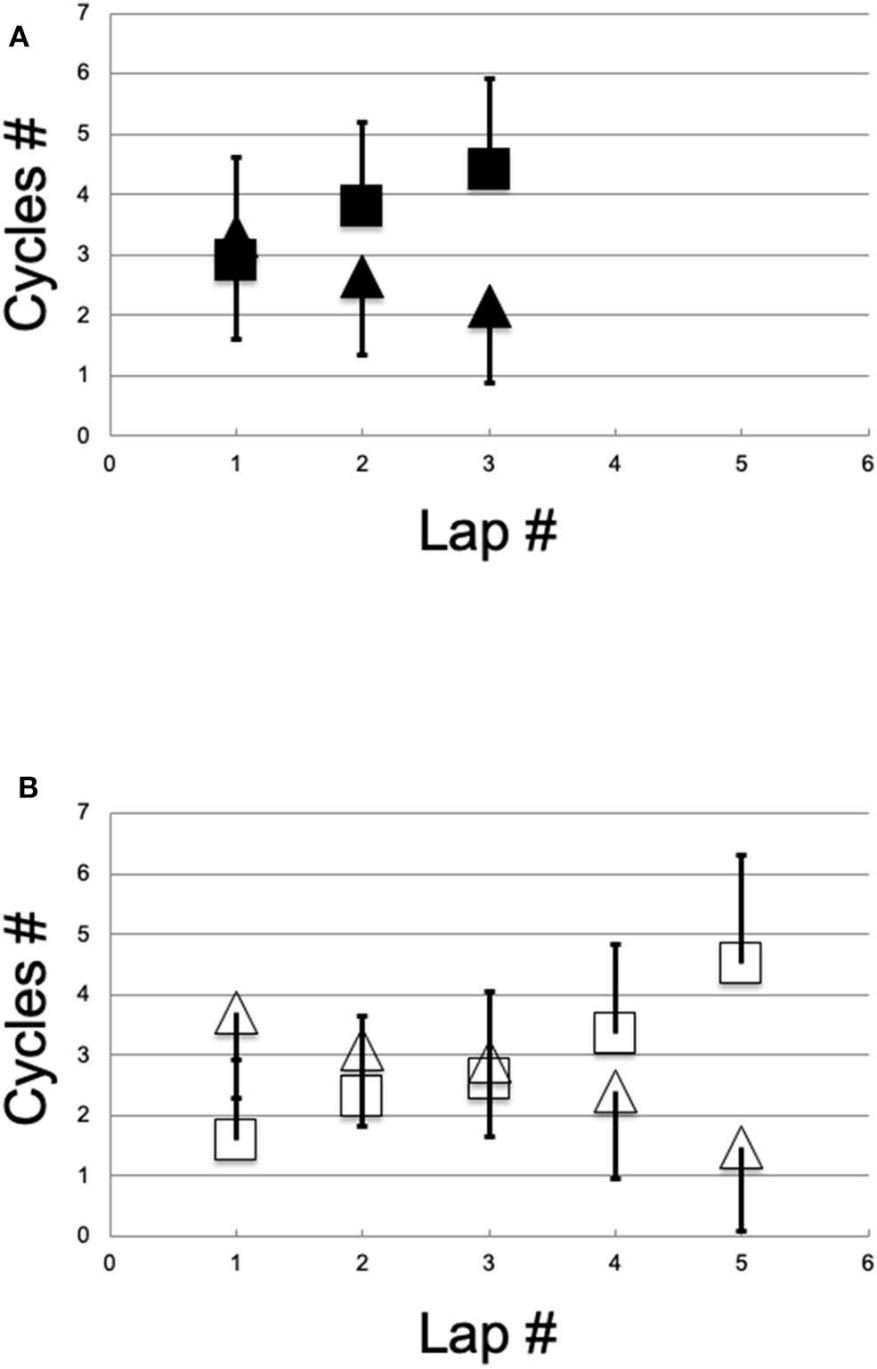
The number (Cycles #) of cycles in relationship to the number of laps (Lap #) for female **(A)**, filled black symbols and male skiers **(B)**, empty black symbols. Squares, G2 (SD bars shown only upwards for clarity); triangles, G3 (SD bars only downwards).

Along the transition section, only the G2 and G3 techniques were used, with the proportion of G2 increasing and G3 decreasing over subsequent laps [Table 2 and Figure 4; *F*_(2, 48)_ = 49, *P* < 0.001]. Neither sex (*P* = 0.190) nor group effects (*P* = 0.149) were significant (Table 2). Lap x sex interaction was significant [*F*_(1, 48)_ = 7.4, *P* = 0.002], with females preferring G2 more than males in the first lap (*P* = 0.027), but no difference in the middle and final laps (*P* = 0.051 and *P* = 0.251). For males, the proportion of G2 significantly increased across the laps (all *P* < 0.001), whereas for females, only the final lap demonstrated higher use of G2 compared with the first. However, lap x group interaction was not significant (*P* = 0.700).

## Discussion

The present study investigated the effect of prolonged ski skating racing on choice of technique in a flat-to-uphill transition section among female and male high-level skiers. Regarding the initial study hypothesis on transition section speed, this speed was shown to be correlated with overall race performance in all skiers. Males achieved faster speeds (albeit not significantly) than females, especially in the first and middle laps (Table 2). The outcome of this comparison confirmed the suitability of our choice to compare sexes over different race lengths while not analyzing the same laps (e.g., first, second, and third laps for both females and males) but rather laps representative of similar levels of fatigue (i.e., first, middle, and final laps representative of rested, average fatigue, and high fatigue, respectively). As a consequence, it was found that males had a higher speed than females when both sexes were rested or had an average level of fatigue (first and middle laps) and the same speed when *maximum* fatigue affected all skiers (last laps). Predictably, elite skiers achieved faster speeds than national (Table 2). Mean section speeds decreased across laps in all skiers due to reductions in both CL and CR, with males showing longer but less frequent cycles than females over the race and elite skiers showing longer cycles over each lap and more frequent cycles only in the final lap. The reduction in CL might be due to some decline of capability to apply propulsive force over the laps (Bellizzi et al., 1998). Specifically, the reduction in CL affected females more than males. This may be explained on the basis of established knowledge of the lower muscular strength and power available to females compared with males (Sandbakk et al., 2014; Stöggl et al., 2018b). By contrast, mechanical internal work is related to CR (Nardello et al., 2011) and a reduction in this kinematic variable might be due to some decline of capability to develop this form of mechanical work across laps.

Regarding the initial study hypotheses on technique choice, all skiers chose only the G2 and G3 techniques with the proportion of G2 increasing, and G3 decreasing, across laps. Elite skiers demonstrated only a non-significant trend of choosing G3 over G2 compared with national skiers. Females preferred G2 more than males only in the first lap, and males continuously increased G2 use across laps, whereas females chose G2 more often than G3 only between the initial and final lap (Table 2 and Figure 4).

The validity of the above-mentioned relationship between choices of force/speed/technique, previously shown in a skating sprint simulation (Andersson et al., 2010) and distance racing using the classical style (Welde et al., 2017; Stöggl et al., 2018b), was confirmed by the results of the present study. The current investigation did not include any metabolic power assessments, thus its findings rely only on kinematics and evaluations of skiers' behavior. Yet it is reasonable to assume a strong link between metabolic power and kinematics and skiers' choice of technique. Similar to the present study, G2 (slower technique) becomes less energy-demanding than G3 (faster technique) as distance or race duration is increased (Kvamme et al., 2005), thus prompting transition to this technique (at the price of a speed decrease), as was effectively the case for the current skiers.

We made use of a video recording-based approach and focused on the flat-to-uphill transition section of the course. Namely, the performance on the inclined section was confirmed to correlate well with that of the overall race (Stöggl et al., 2018a) and a video analysis was adequate for the measurements needed for the study. Nevertheless, future more detailed on-snow studies of skiers' behavior over real races could benefit from using—as a more effective alternative—wearable technologies (Marsland et al., 2017). Such studies could overcome the limit of this study of only measuring over one section of the course. Another limitation of this study is that it focused only on senior skiers. Further studies could involve youth and elderly skiers (Nikolaidis and Knechtle, 2018; Nikolaidis et al., 2018).

Both section and mean lap speed decreased over the distance, i.e., all racers showed a positive pacing. Although not significantly, elite skiers confirmed a more even pacing than national skiers (Table 2; Karlsson, 2017; Stöggl et al., 2018a, 2020). Furthermore and as already documented for the classical style, females also have a considerable margin for speed improvement in flat-to-uphill ski skating (Stöggl et al., 2018a).

Over a long-distance skating event, it was found that race performance was associated with speed and the use of G3 technique along a flat-to-uphill transition section similarly to previous findings regarding a simulated sprint time trial (Andersson et al., 2010) and generic XCS races (Stöggl et al., 2018a). However, compared to Bilodeau et al. study 1996, the current male skiers participated in a similarly long race and were recorded over a similarly steep section of the course. Further, the skiers in this study only partially preferred G2, i.e., particularly only in the final lap. elite skiers preferred G2 less than national, albeit not significantly (Table 2, Figure 4). A further relevant difference is that the current 2018 skiers and equipment were probably better than those of Bilodeau et al. (1996) and Pellegrini et al. (2018). Regarding sex differences, the above hypothesized females' potential speed improvement margin in flat-to-uphill skating might be achieved by training to extend CL and to prefer G3 to G2 especially when rested (i.e., at the beginning of the training session).

Prolonged ski racing clearly elicited profound fatigue effects in the skiers analyzed. The present study showed a race speed decrease over the laps, paralleled by both CL and CR reductions (Table 2). Kim et al. (2017) showed a decrease over a simulated 12-km roller skiing race using the double poling technique. When considering double poling similar to G3 (because both techniques require parallel upper-limb pushes supporting ski gliding), Kim et al. (2017) findings are akin to the present study regarding race speed decrease and its determinants. Ohtonen et al. (2018) showed how a simulated 20-km race in skating style worsened final sprint performance using G3. Namely, after the simulated race, speed and CR were significantly lower, whereas CL decreased only slightly. Prolonged skiing (i.e., exercising from 1 to 24 h) clearly places a sustained burden on the metabolic machinery (Davies, 1981). Regarding the available metabolic power, the best performing skiers typically have higher *maximum* oxygen consumption (VO_2Max_) than slower skiers (Holmberg, 2015). In addition to that, less successful skiers can rely also on a lower average fraction of their available VO_2Max_ that is acknowledged to decrease with increasing effort time (Davies, 1981). Moreover, it is interesting to notice that the G2 technique looks a little similar to the preferred, and less metabolically demanding, technique on flat courses, G4 (Bilodeau et al., 1996). Boldt et al. (2016) showed that G4 induces a spontaneous locomotion–respiration entrainment allowing an oxygen consumption saving of about 4% in a roller skiing model. By inference, it may be that, when fatigued, skiers switch from G3 to G2, also to cope with reduced available metabolic power. Then the extra metabolic demand due to switching from G3 to G2 could be at least partially compensated by a saving in breathing effort.

## Conclusions

In long distance ski skating, the speed in transition sections was shown to correlate with overall race performance in all skiers. Mean section speeds decreased across laps in all skiers due to reductions in both cycle length and rate. All skiers chose only the G2 and G3 techniques with the proportion of G2 increasing, and of G3 decreasing, across laps, with the best-ranked skiers showing only a non-significant trend of choosing G3 more than G2, compared with the worst-ranked skiers.

## Data Availability Statement

The datasets generated for this study are available on request to the corresponding author.

## Ethics Statement

The studies involving human participants were reviewed and approved by Norwegian Center for Research Data. The patients/participants provided their written informed consent to participate in this study.

## Author Contributions

All authors listed have made a substantial, direct and intellectual contribution to the work, and approved it for publication.

## Conflict of Interest

The authors declare that the research was conducted in the absence of any commercial or financial relationships that could be construed as a potential conflict of interest.
